# Prescriptions and Dispensing

**Published:** 1908-10-17

**Authors:** 


### Therapeutics.

### PRESCRIPTIONS AND DISPENSING.

### OINTMENTS

Though the practitioner will probably obtain most ointments ready prepared, there are occasions, especially in the country, when he may need to make his own. In concluding our general remarks about ointments and the dispensing of them, the following precautions may prove useful:—

When heat is employed, it is a safe rule never to heat more than is essential; a water-bath should always be employed, and not the direct heat of a flame. An exception must be made in the case of Unguentum Hydrargyri Nitratis, in preparing which a sand-bath is ordered to be used, and a temperature of 290° F. is required. This, however, is rather a matter for the manufacturing pharmacist than for the dispensing doctor.

During cooling more or less separation of the ingredients of an ointment that has been made by heat may occur; to obviate this it is usually needful to stir constantly during the cooling.

The spatula used in ointment-making should usually be of bone, horn, or vulcanite. A flexible steel spatula, though more convenient sometimes, should never be employed when there is a possibility of the iron being acted on by one of the ingredients. Vegetable extracts very frequently contain tannin and a small amount of some organic acid, and such extracts are darkened to a greater or less degree if manipulated with a steel spatula. If no moisture is present this action does not occur, and an ointment containing tannic acid itself is not darkened by a steel spatula if all the ingredients are free from moisture; but it is better to be on the safe side.

Salicylic acid is even more readily discoloured by iron than is tannin; and when it, or a salicylate, is an ingredient of an ointment, steel must be avoided. Salts of mercury are attacked by iron with the liberation of metallic mercury, if a vehicle is present which permits them to come into reaction. With a dry, fatty basis the change does not occur.

We will conclude with a few brief notes upon some branches of dispensing with which the practitioner should be familiar, though he will not need to use his knowledge of them practically.

### CAPSULES.

Capsules, though sometimes used for powders, are more frequently used for liquids or soft semi-liquid substances. The object is the same as in cachets—namely, to enclose the medicament in a receptacle which can easily be swallowed whole, and which will readily dissolve in the stomach without itself having any therapeutic activity.

The principal material of which capsules are made is gelatin; gum also enters largely into the composition of some. Soft gelatin capsules are the commonest; they are manufactured in large quantities by machinery.

The first step in making up capsules is to prepare the mass of which the envelope itself is to consist. The following is a suitable formula:—

| | |
|---|---|
| Gelatin ... ... ... ... ... | 30 oz. |
| Water ... ... ... ... ... | 50 oz. |

The gelatin is soaked until all the water has been absorbed, and then are added:—

| | |
|---|---|
| Glycerin ... ... ... ... ... | 15 oz. |
| Acacia mucilage ... ... ... ... | 7½ oz. |

the whole being heated, with gentle stirring, on the water-bath until uniform throughout.

The moulds on which the empty capsules are to be made consist of solid oval pieces of metal, rounded at one end, but prolonged at the other into a thin stem an inch or more in length. A number of these are attached to a flat disc of wood with a handle, in much the same way as a number of pills stuck on needles are attached to one cork for gelatin coating. Before being used, each mould is wiped with a clean but oily cloth, which leaves a very thin film of oil on its surface to prevent the gelatin mass sticking.

A sufficient quantity of the mass having been melted in a water-bath, which should be considerably below boiling temperature, the surface of the mixture, which is usually more or less frothy, is skimmed to one side, and the moulds are immersed in the gelatin mixture until the surface is a quarter of an inch or rather less up the stems. They are then slowly withdrawn, excess of the molten mass being allowed to drain off; they are then inverted, and kept turned upwards until the mass on the moulds has set. This does not take long. As soon as cooling has occurred, each capsule can be slipped off its mould, the elastic nature of the material allowing the narrow neck to slip easily over the thicker part of the metal. The necks of the capsules are then trimmed almost away with scissors, and they are ready for filling. Shrinkage occurs as the capsules dry.

If dry powders are to be placed in the capsules, they are prepared and weighed out exactly as we have described for papers or cachets. Each capsule in turn is attached to a small funnel by inserting the stem of the latter for a short distance into the open end, and the powder is shaken in through the funnel.

As a rule, however, the required medicament is a liquid, or a thin paste, and in such cases a sufficiency of it is put into the barrel of a glass syringe, and the capsules in turn are slipped on to the nozzle and filled by pressure on the piston.

Care has to be taken not to quite fill the capsules, but to leave a small empty space at the top; otherwise it is not possible to seal them off properly. The sealing can be done by touching the mouth of each in turn with a hot glass rod dipped in some of the original molten matrix; the hot rod just melts the edges of the capsule, and the small quantity of gelatin mixture put on forms a stopper which, on cooling, is continuous with the walls.

It is clear that capsules of this kind cannot be used for aqueous liquids, nor for any others that will dissolve gelatin. Oily liquids are best, and dry, or nearly dry, substances can be made into a thin paste by mixing with a little oil, and thus introduced into the capsule.

